# Feedback of action outcome retrospectively influences sense of agency in a continuous action task

**DOI:** 10.1371/journal.pone.0202690

**Published:** 2018-08-23

**Authors:** Hiroyuki Oishi, Kanji Tanaka, Katsumi Watanabe

**Affiliations:** 1 Faculty of Science and Engineering, Waseda University, Tokyo, Japan; 2 Research Center for Advanced Science and Technology, The University of Tokyo, Tokyo, Japan; 3 Japan Society for the Promotion of Science, Tokyo, Japan; University G d'Annunzio, ITALY

## Abstract

Here, we investigated whether explicit feedback on the result of the action (success or failure) modified sense of agency (SoA) in a continuous action task. Participants carried a white dot with a delay to a target square while avoiding obstacle squares. The color of the target changed unpredictably between white and blue. A trial was considered as successful or failed if the dot reached the target while it was white or blue. Thus, actions during the task resulted in almost identical experiences of successful and failed trials. After each trial, the participants reported to what extent they felt that they had been in control of the dot. The results showed that SoA was higher with shorter delay (i.e., easier control) and in the successful trials. These findings indicate that the sense of online control and the evaluation of continuous action based on feedback independently influence SoA. Particularly, the evaluation retrospectively modulated SoA.

## Introduction

Sense of agency (SoA) is a subjective feeling that one is controlling one’s own actions and one’s action causes changes in external circumstances [1–2]. Numerous studies have shown that SoA can be augmented or attenuated by internal motor signals and/or external cues [3–4] with tasks involving a simple action [5–6] and a continuous action [7–8]. SoA can be measured in various ways [5,9–10]. As an example of explicit measurement of SoA, after participants finished a given task, they answered to what extent they caused the external event by either verbal report or use of computer mouse [5,9]. As an example of an implicit measurement of SoA, they reproduced a delay between action and a perceived outcome [10].

The comparator model (a computational model of motor control) suggests that SoA basically arises from internal cueing and is determined by a match between a sensory prediction of an action’s consequence and the actual consequence [11–12]. If the prediction and the actual result match, the sensory event is likely to be recognized as generated by the agent, resulting in a strong SoA. In contrast, if the predictions do not match, the event is regarded as an external event, resulting in a weak SoA. However, contrary to the comparator model, some studies have shown that SoA could arise in the absence of internal motor signals, that is, exclusively through external cues such as priming [13–14]. For example, Wegner and colleagues showed that SoA can arise even when participants do not move [14]. In their experiment, a participant watched himself/herself and a helper participant through a mirror. The helper, who was behind the participant, performed a series of movements with his/her arms, while the participant never moved. The participant heard instructions to engage in the movements made by the helper before commencement of actual movement. Then, the participant felt SoA for the movements by the helper. Moreover, as SoA is modulated by both internal motor signals and external cues [3–4], Moore and Fletcher proposed a cue integration theory (see also the “two-step model” of agency [13]). In this theory, SoA can be regarded as posterior (according to the Bayesian cue integration framework) and in particular, as determined by the integration of various cues, internal cues (e.g., effort, prediction of reward and knowledge) and external cues and their reliability. This was supported by studies in which when the reliability of internal motor information was low, the influence of external cues to SoA increased [15].

In the present study, we were interested in how SoA emerges during continuous action [7–8]. In daily life, people are always required to do continuous actions such as driving a car or doing sports. In this situation, they may need to prepare for the next movement while they are processing feedback of the movement just completed, which likely leads to a situation where a precise comparison of the predicted result of an action and corresponding feedback is more difficult [16] than a task with a simple action. Therefore, it might be assumed that during continuous action, external cues have more impact on SoA than internal cues compared to a simple action.

Indeed, Wen and colleagues did show that SoA during continuous action was influenced by not only the action–feedback association (i.e., internal cue) but also by goal-directed inference (external cues), which represents high-level cognition arising from the fact that the participant was able to achieve a given task [8]. Their results suggested that if internal cues were unreliable, external cues affected SoA more than the internal cues did, a finding related to the cue integration theory [17]. In their experiment, participants were required to control a dot and carry it to a target by key press. When they pressed the left or right key, the direction of the dot shifted counterclockwise or clockwise by 10°. Two conditions were prepared: self-controlled and computer assisted-conditions. In the former, the dot always reacted to the key press, while in the computer assisted-condition, the key press was ignored when the key press ordered a change of dot direction toward the side opposite the target (i.e., an indication of computer assistance), because such a change was regarded as a roundabout way. Note that the participants were not informed of the computer assisted condition. After each trial (i.e., the dot reached the target), participants were asked to what extent the dot was under their control, which represented indices of SoA. They showed that SoA, reaching time, and the number of key presses were respectively higher, shorter, and smaller in the computer-assisted condition than in the self-controlled condition, implying that better performance by the assistance led to higher SoA even if the participant’s key presses were sometimes ignored. That said, as the participants were not notified about the computer assistance and results of interviews about the awareness were not reported, the study could not discriminate those who understood the assistance intervention from those who mistakenly understood the good performance to be owed to them. Embarking from these findings, Inoue and colleagues presented a message informing the participant of the experimental condition (computer-assisted or non-assisted) before each trial [18]. Nevertheless, SoA remained greater in the computer-assisted condition than in the non-assisted condition, showing that goal-directed inference influenced SoA more than action–feedback association. Since the participants did not receive feedback on their task performance in these experiments, SoA was based on a self-evaluation of reaching time and the number of key presses after each trial. Although previous studies also showed that better task performance by self-evaluation contributes to higher SoA [7,8], the process or perceived effort (i.e., internal cues) and the result (i.e., external cues) could not be completely dissociated, because more effort probably led to a better result. Therefore, it remains unclear how task performance by self-evaluation and objective evaluation of the task performance (i.e., feedback of a result) respectively influence SoA during continuous action.

In the present study, we aimed at isolating the effects of feedback of results on SoA. To do so, we adopted the dot control task [8,18], but modified the following points. First, participants moved the dot to a target whose color altered between white and blue with random intervals. The trial was successful or failed when the dot arrived at the target while the color was white or blue; that is, the result feedback changed unpredictably between success and failure even though task performance was almost identical. Second, obstacles were placed, and the trial also ended as a failure when the dot entered the obstacle. This allowed modulation of task difficulty. Third, the computer-assisted condition was not adopted because we did not purpose to investigate effects of the assistance on SoA. Following Inoue et al., SoA is expected to become stronger in a better performance even when participants understand that their better performance is due to the computer assistance, which indicates that goodness of performance has an impact on generation of SoA [18]. According to cue integration theory [17], SoA relies more on external cues when internal cues are not reliable. Hence, in the present study, if only goodness of actual performance influences SoA (i.e., self-evaluation for reaching time and the number of key presses), result feedback would not likely influence SoA. Alternatively, if the feedback itself influences SoA regardless of task performance, SoA should be higher with feedback of success than with that of failure.

## Method

### Participants

Twenty-two volunteers (12 male, 10 female; mean age = 21.18 years, standard deviation = 1.19) participated in the experiment. All participants had normal motor function and were naïve to the purpose of this study. This experiment was approved by the institutional review board (IRB) of Waseda University (2015–033), and conducted in accordance with the ethical standards in the 1964 Declaration of Helsinki. Written informed consent was obtained from all participants in advance.

### Stimuli and procedure

**Fig 1:**
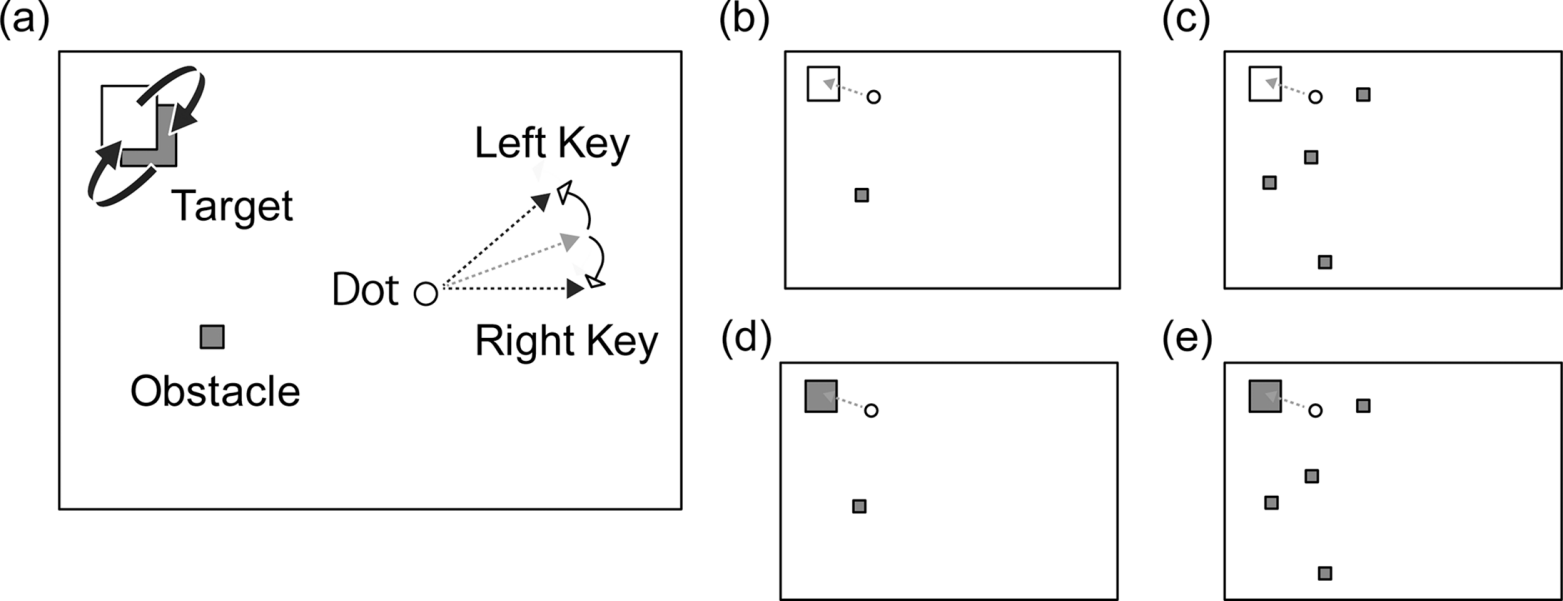
The schematic illustration of each condition (conditions varied by feedback and obstacle(s)). Note that the delay condition was not illustrated here. The movement direction of the white dot is depicted with an arrow. The color of the target changed with random intervals from 500 to 2000 ms. Note that the grey squares in the figure were blue in the experiment. (a) Names of each component. (b) A successful trial in the one obstacle condition. (c) A successful trial in the four obstacles condition. (d) A failed trial in the one obstacle condition. (e) A failed trial in the four obstacles condition.

We prepared three types of stimuli; dot, target, and obstacle (Fig 1A). At the beginning of each trial, a white dot (4.9 mm diameter) appeared 114 mm away from the center of the screen (a 23-inch LCD monitor) on the horizontally opposite side from the target on a black background. The white dot moved in a fixed speed (106 mm/s). In each trial, the initial movement direction of the dot was randomly determined, but it was not toward the target side. That is, when the target was presented at left, the initial direction of the dot was toward the upper- or lower-right area, and when the target was at right, the dot initially moved toward the upper- or lower-left area. Please note that, after data collection, we found that when the target was at right the dot moved toward only the upper-left area, not the lower-left area, due to an error in the program code. That said, as this discrepancy occurred in all conditions, we believe that it did not distort the results. If the dot reached the edge of the screen, its movement changed corresponding to the incidence and reflection angles against the border. At any time, if the left or right key was pressed, the dot turned 20°counterclockwise or clockwise. The dot did not change direction even if the key was kept pressed down; that is, discrete and repeated key presses were required to continuously change the direction. The target was a square (27 mm × 27 mm) whose color alternated between white and blue at a randomly determined interval from 500 to 2000 ms. The target was randomly located at one of four possible locations (57.8 mm diagonally from a corner toward the center of the target). The obstacle was a blue square (8.2 mm × 8.2 mm) randomly placed on the left or right side of the screen (with the dot then placed on the other side).

Two independent variables were prepared to modulate task difficulty: delay and number of obstacles. Two types of delay between key press and the reaction of the dot were prepared: 250 ms and 750 ms, which was fixed within a trial. In addition, the number of obstacles was determined for each trial: one or four (Fig 1B–1E).

**Fig 2:**
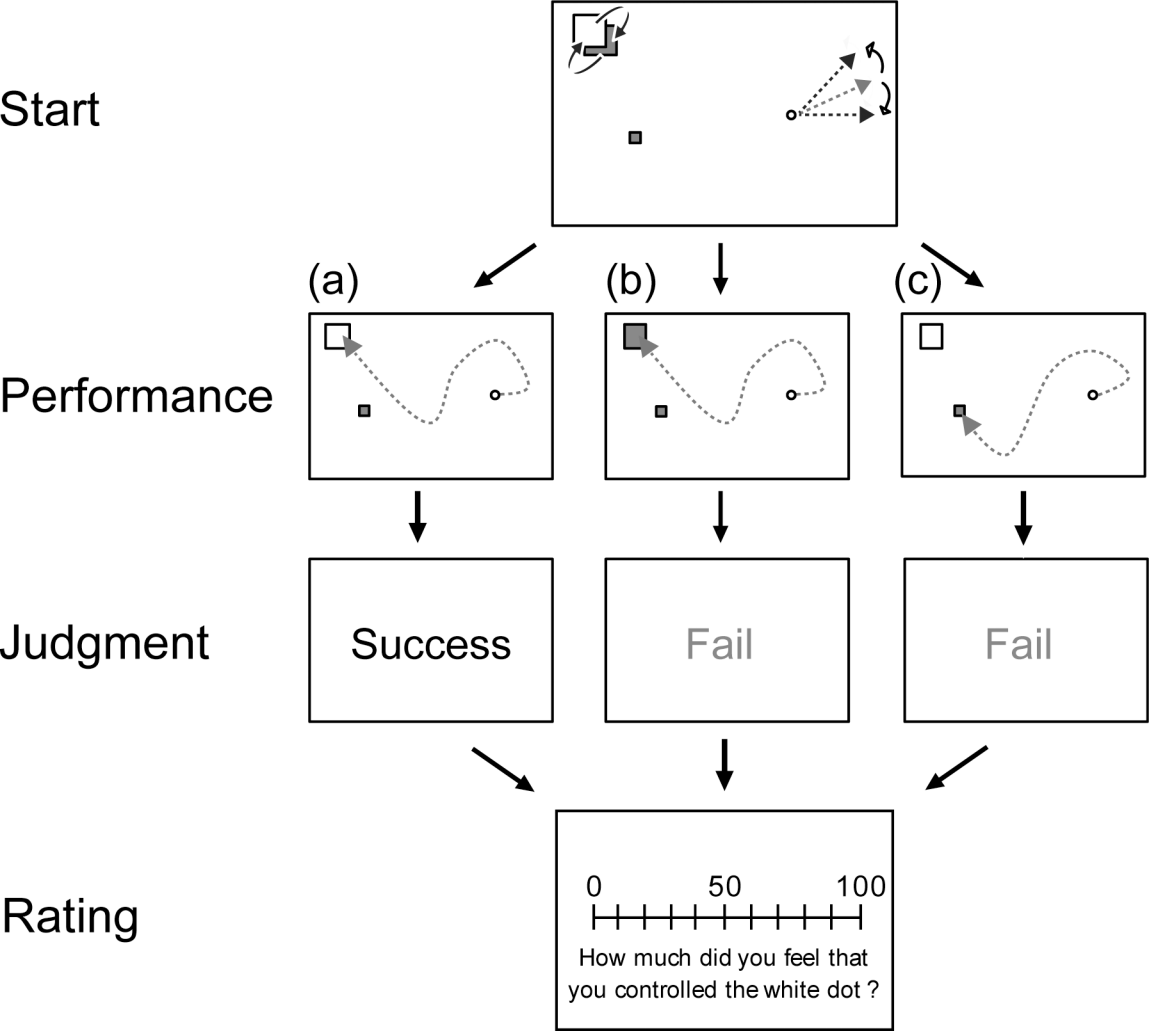
The schematic illustration of the experiment. After performance of the task, feedback on the result (i.e., judgment) was shown and the participant self-rated their SoA. (a) The flow of a successful trial. (b) The flow of a failed trial. (c) The flow of a trial that failed due to entry into an obstacle.

The participants were instructed to move the white dot to the target as quickly as possible and reach the target when the target’s color was white (successful trial; Fig 2A) and not blue (failed trial; Fig 2B). Alternatively, the trial ended as a failure when the dot entered the obstacle (Fig 2C). No time limit was set. After the participant succeeded or failed, feedback (“Success” or “Fail”) was shown at the center of the screen for 1200 ms; then, the participant used a computer mouse to report to what extent they felt that they had been in control of the white dot in a visual analog scale (0–100). The participants were informed beforehand that a delay might occur between key press and the reaction of the dot or that an order by key press might be ignored (in fact, the key press was always reacted with a certain delay), which was identical to the instructions adopted in the previous study [8]. Since the previous study investigated the effects of computer-assistance on SoA, they informed the participants of the ignorance of key presses. This was not relevant to that in the present study, as we only aimed to focus on effects of feedback on SoA. But, here, we tried to adopt the experimental procedure used in the previous study as much as possible we could. There were four conditions: Two delay conditions (250 ms or 750 ms) × Two obstacle conditions (one or four obstacles). Each condition had 30 trials, resulting in 120 trials in total. The order of the trials was randomized. The participants were allowed to take a short break every 20 trials.

### Data analysis

We independently measured reaching time, the number of key presses, and rating of SoA. For the number of trials in each judgment type (successful judged trial, failed judged trial, or failed trial by entry to an obstacle), we firstly conducted one-way analysis of variance (ANOVA) with judgment type as a within-subject factor. In post hoc tests, Shaffer’s method was used where appropriate. Cohen’s *d* was used for the two-sample *t*-tests (two-tailed). For rating of SoA, we conducted three-way analysis of variance (ANOVA) with judgment type (successful or failed), two types of delay (250 or 750 ms), and the number of obstacles (one or four) as within-subject factors. Effect sizes (η_*p*_^2^) were calculated for all of the ANOVAs.

## Results

The mean number of successful and failed judgment trials out of 120 trials was 49.86 (*SD* = 7.41) and 48.05 (*SD* = 5.57), respectively. The mean number of failed trials by entry into obstacles was 22.09 (*SD* = 8.54; Table 1). For the number of assigned trials, a one-way ANOVA revealed a significant main effect between successful and failed trials (*F*(2, 42) = 66.87, *p* < 0.001, η_*p*_^2^ = 0.76); then, post-hoc multiple comparisons with Shaffer’s method showed that the mean number of successful and failed judgment trials were larger than that of failed trials by entry to obstacles (*t*(21) = 9.85, *p* < 0.001, *d* = 3.47; *t*(21) = 8.69, *p* < 0.001, *d* = 3.60) while the number of successful and that of failed judgment trials were not significantly different (*t*(21) = 0.85, *p* = 0.40). This indicated that the present experimental paradigm did not allow participants to precisely control the dot right before entry to the target; therefore, we can assume that the efforts before entry to the target were controlled between the successful and failed trials.

**Table 1:** The mean number of trials in each condition (standard deviation).

	250ms delay		750ms delay		
	1 obstacle	4 obstacles	1 obstacle	4 obstacles	Sum
Successful judgment trials	14.27 (2.47)	12.68 (3.16)	13.91 (2.58)	9.00 (2.74)	49.86 (7.41)
Failed judgment trials	14.09 (2.68)	11.64 (3.03)	12.55 (2.99)	9.77 (3.02)	48.05 (5.57)
Failed trials by entry into obstacles	1.64 (1.50)	5.68 (3.34)	3.55 (2.20)	11.23 (3.44)	22.09 (8.54)

For the mean number of failed trials by entry into obstacles (i.e., 22.09), we performed a 2 (delay: 250 or 750 ms) × 2 (obstacle: one or four) repeated-measures ANOVA. We found significant main effects of delay and obstacle (*F*(1, 21) = 68.37, *p* < 0.001, η_*p*_^2^ = 0.76; *F*(1, 21) = 145.61, *p* < 0.001, η_*p*_^2^ = 0.87). The interaction between delay and obstacle was also significant (*F*(1, 21) = 33.37, *p* < 0.001, η_*p*_^2^ = 0.61). The post-hoc tests showed that the number of failed trials in the four obstacles condition was significantly larger in the delay with 750 ms than with 250 ms (*F*(1, 21) = 75.79, *p* < 0.001, η_*p*_^2^ = 0.78) and that in the delay with 750 ms was larger in the four obstacles condition than in the one obstacle condition (*F*(1, 21) = 202.29, *p* < 0.001, η_*p*_^2^ = 0.90). These results indicate that the difficulty of the present task (i.e., the number of failed trials by entry into obstacles) was modulated by delay and the number of obstacles, and in particular, control in the delay with 750 ms and four obstacles is most difficult among the experimental condition. As the present study focused on the successful and failed judgment trials, we excluded the failed trials by entry into obstacles from the next analysis.

### Rating of SoA

**Fig 3:**
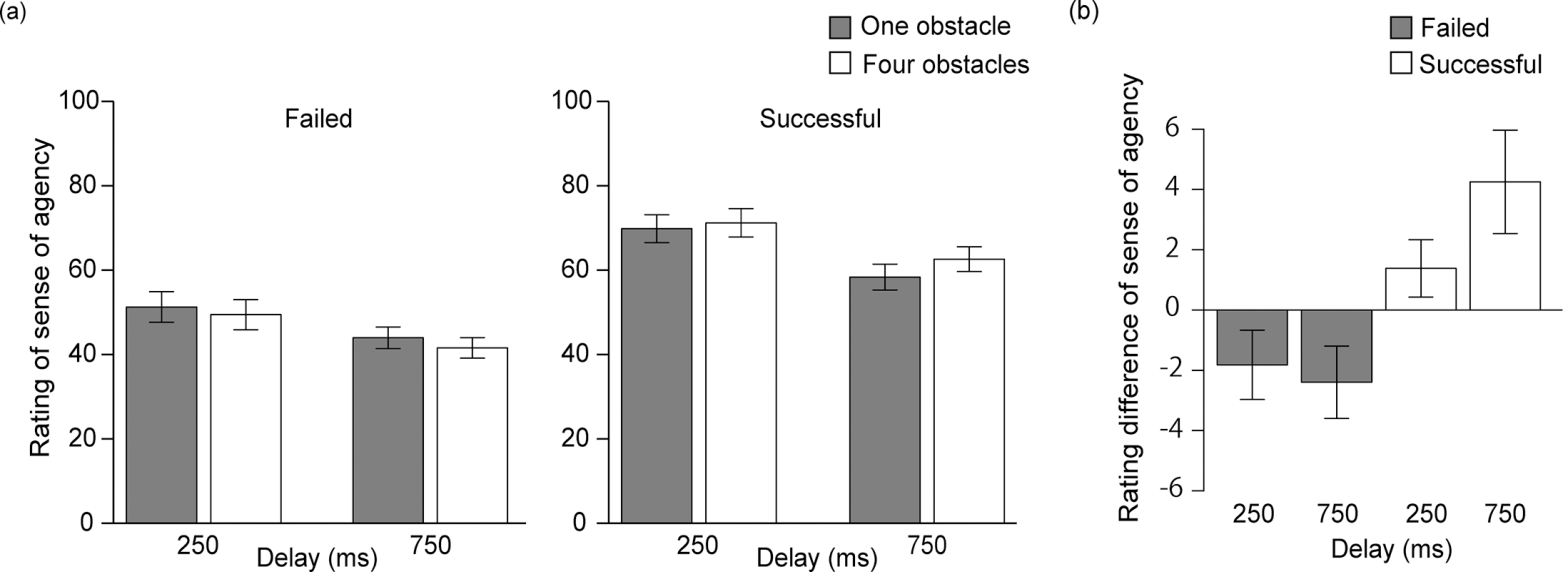
Results of experiment. (a) The mean rating of SoA. (b) Rating difference: SoA with four obstacles subtracted from SoA with one obstacle in each condition. Error bars indicate the standard errors of the mean.

For the average of self-rated SoA, we conducted a 2 (successful or failed trials) × 2 (delay: 250 or 750 ms) × 2 (obstacle: one or four) repeated-measures ANOVA (Fig 3A, see S1 Table for individual information in each experimental condition). The main effect between successful versus failed trials was significant (*F*(1, 21) = 45.71, *p* < 0.001, η_*p*_^2^ = 0.69). This indicates that the feedback of results (success or failure) affected SoA. The main effect of delay was also significant (*F*(1, 21) = 16.46, *p* < 0.001, η_*p*_^2^ = 0.44), which is consistent with the previous studies showing that a longer delay led to lower SoA [4,19–20]. In contrast, the main effect of obstacle was not significant (*F*(1, 21) = 0.27, *p* = 0.61), indicating that the number of obstacles (one or four) did not have a concrete influence on SoA. However, the interaction between trial success and obstacle was significant (*F*(1, 21) = 18.41, *p* < 0.001, η_*p*_^2^ = 0.47); post-hoc comparisons revealed that in the successful trials, SoA was higher for four obstacles than for one (*F*(1, 21) = 10.56, *p* < 0.01, η_*p*_^2^ = 0.33), and the reverse was true for the failed trials (*F*(1, 21) = 5.35, *p* < 0.05, η_*p*_^2^ = 0.20; Fig 3B).

### Reaching time and number of key presses

**Fig 4:**
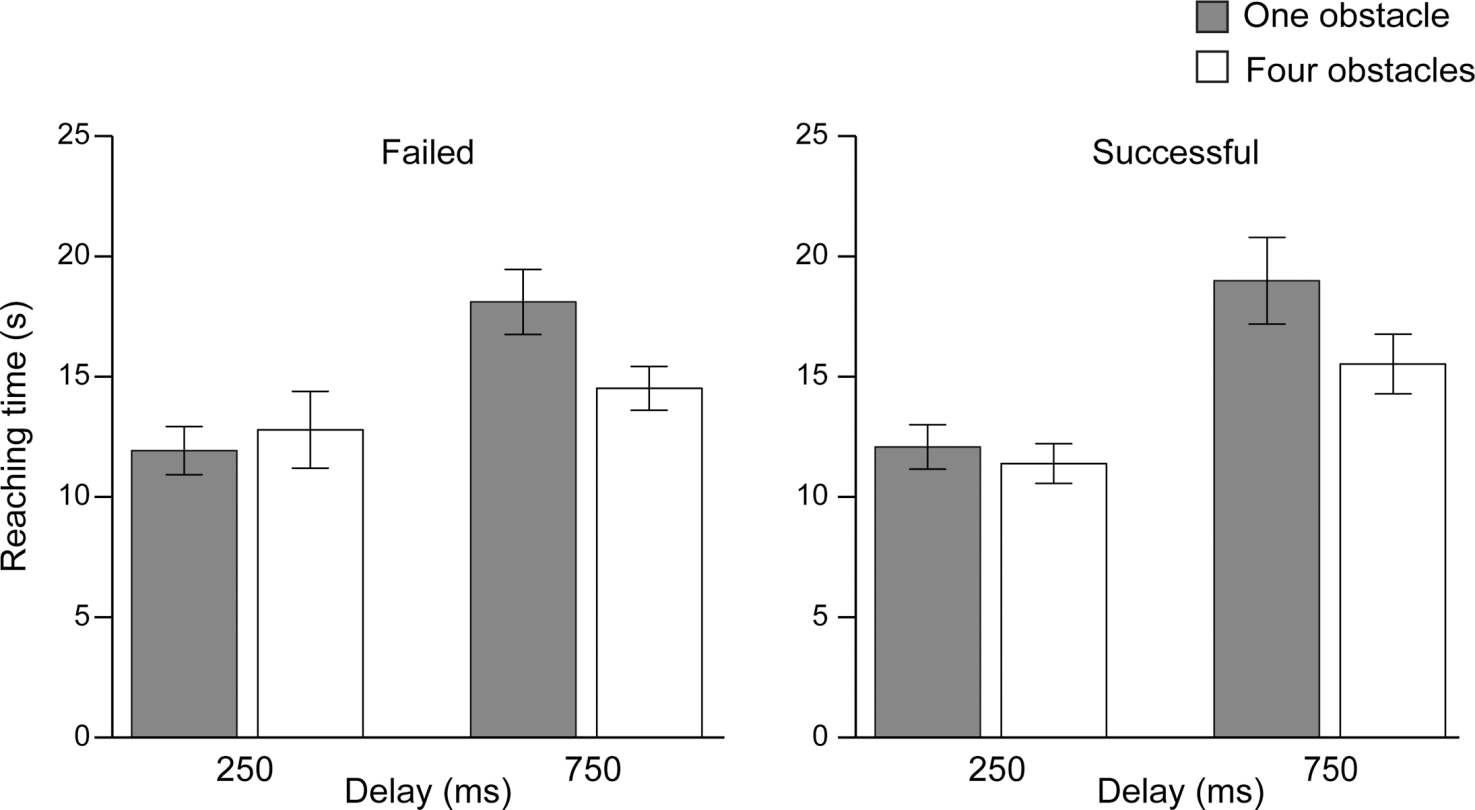
The reaching time in each condition. Error bars indicate the standard errors of the mean.

For the average of reaching time, we conducted a 2 (successful or failed trials) × 2 (delay: 250 or 750 ms) × 2 (obstacle: one or four) repeated-measures ANOVA (Fig 4). We did not find a significant main effect between successful and failed trials (*F*(1, 21) = 0.13, *p* = 0.71), indicating that the reaching time did not vary by target color. The main effects of delay and obstacle conditions were significant (*F*(1, 21) = 44.20, *p* < 0.01, η_*p*_^2^ = 0.68; *F*(1, 21) = 9.01, *p* < 0.01, η_*p*_^2^ = 0.30), indicating that delay and the number of obstacles affected the difficulty of the task. The interaction between delay and obstacle was also significant (*F*(1, 21) = 7.14, *p* < 0.05, η_*p*_^2^ = 0.25), while the other interactions were not significant (judgment × delay, *F*(1, 21) = 1.90, *p* = 0.18; judgment × obstacle, *F*(1, 21) = 0.67, *p* = 0.42; judgment × delay × obstacle, *F*(1, 21) = 0.76, *p* = 0.39). Post-hoc comparisons showed that the reaching time was significantly shorter in the delay with 250 ms than in the delay with 750 ms both when the number of obstacles was one and four (*F*(1, 21) = 60.25, *p* < 0.001, η_*p*_^2^ = 0.74; *F*(1, 21) = 7.03, *p* < 0.05, η_*p*_^2^ = 0.25). With 750 ms delay, the reaching time was significantly longer in the one obstacle condition than in the four obstacles condition (*F*(1, 21) = 10.21, *p* < 0.001, η_*p*_^2^ = 0.33) while with 250 ms, it was not significantly different (*F*(1, 21) = 0.02, *p* = 0.88).

**Fig 5:**
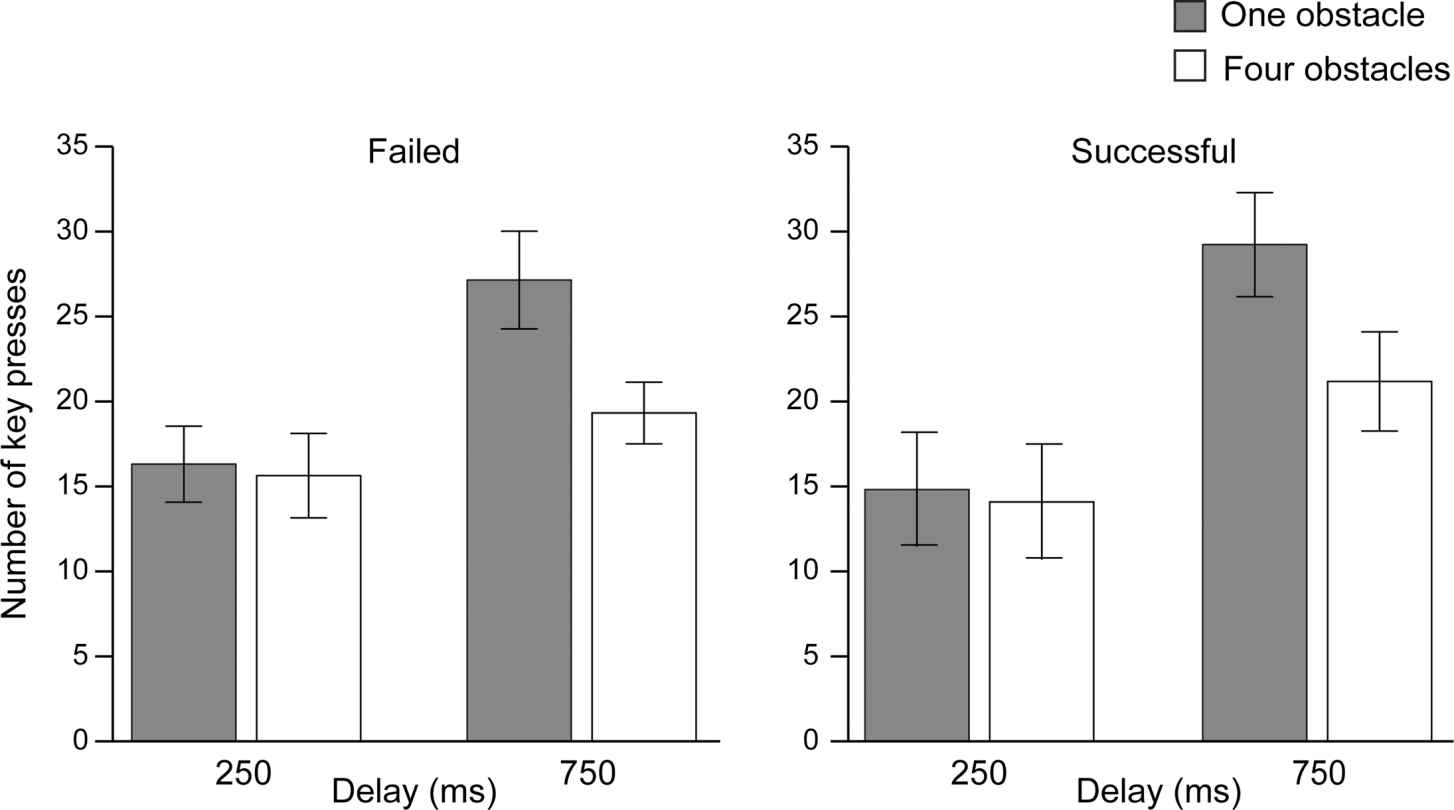
The total number of key presses in each condition. Error bars indicate the standard errors of the mean.

We conducted the same analysis for the number of key presses as for reaching time (Fig 5): a 2 (successful or failed trials) × 2 (delay: 250 or 750 ms) × 2 (obstacle: one or four) repeated-measures ANOVA. We did not find a significant main effect between successful and failed trials (*F*(1, 21) = 0.04, *p* = 0.85), indicating that the number of key presses did not change regardless of whether the target reached successfully or not. The main effects of delay and obstacle were significant (*F*(1, 21) = 43.97, *p* < 0.001, η_*p*_^2^ = 0.68; *F*(1, 21) = 21.50, *p* < 0.001, η_*p*_^2^ = 0.50), indicating that delay and number of obstacles both affected the difficulty of the task. The interaction between delay and obstacle was significant (*F*(1, 21) = 8.45, *p* < 0.01, η_*p*_^2^ = 0.29), while the others were not (judgment × delay, *F*(1, 21) = 1.94, *p* = 0.18; judgment × obstacle, *F*(1, 21) = 0.01, *p* = 0.94; judgment × delay × obstacle, *F*(1, 21) = 0.003, *p* = 0.96). Post-hoc comparisons showed that the number of key presses was significantly larger with the 750 ms than with the 250 ms delay, both with one and four obstacles (*F*(1, 21) = 47.50, *p* < 0.01, η_*p*_^2^ = 0.69; *F*(1, 21) = 8.34, *p* < 0.01, η_*p*_^2^ = 0.28). In the delay of 750 ms, the number of key presses was larger in the one obstacle condition than in the four obstacles condition (*F*(1, 21) = 16.89, *p* < 0.01, η_*p*_^2^ = 0.45), while with the 250 ms delay, it was not significantly different (*F*(1, 21) = 0.45, *p* = 0.51).

Thus, taken together, the main effects of delay and obstacle conditions were significant for both reaching time and the number of key presses. Therefore, we can assume that the present experimental paradigm effectively modulated task difficulty.

## Discussion

In the present study, we investigated whether SoA in the continuous task was influenced by feedback of the final outcome. We found that: (1) SoA was attenuated by longer delay between key press and response. (2) SoA was higher in the successful trials than in the failed trials. (3) The differences of SoA between the successful and failed trials were more pronounced in the condition with more obstacles. These results indicate that SoA in the continuous task was influenced not only by the online comparison between action and outcome during the task but also by the goal-directed inference generated by the feedback of the final outcome. This supports the general notion of cue integration theory [17].

Taking the results in turn, first, SoA was attenuated with longer delay between key press and response, as observed in the previous studies [4,19–20]. Moreover, the reaching time and the number of key presses were respectively longer and larger in the longer delay condition (750 ms). That is, the longer delay made control of the dot more difficult. Interestingly, in the longer delay condition, the reaching time and the number of key presses were respectively shorter and smaller as the number of obstacles increased. On the other hand, in the shorter delay condition, the number of obstacles did not affect the reaching time or the number of key presses. This might suggest that the participants changed their strategy toward the task with the length of delay and the number of obstacles. Particularly in the four obstacles condition with longer delay, their strategy might have moved toward carrying the dot to the target with a smaller number of key presses (they might sometimes wait for a bounce from the edge), while in the other conditions they might try to carry the dot to the target quickly by using small adjustments of movement direction of the dot. Given that, they might not receive sufficient visual feedback of their key presses; therefore, their agency judgments more depended on the feedback in the four obstacles condition with longer delay. That said, as the number of failed trials by entry to the obstacles was significantly larger in both the delay of 750 ms and four obstacles condition, and the failed trials were excluded from the main analysis, the numbers of available trials in the analysis were not equal among the conditions. Therefore, we could not simply conclude the effects of delay on the task performance or strategy. This point needs to be investigated in future studies.

Second, the most important finding in the present study was that SoA was influenced by feedback of the final outcome even though the reaching time and the number of key presses were not significantly different between the successful and failed trials. Van der Weiden and colleagues demonstrated that SoA was low when the feedback did not match the expectation pre-determined by priming or instruction [21] (see also Wen et al. [22]). In the present study, given that we could assume that most participants likely expected a successful feedback before they started a trial, the low rating of SoA in the failed trials partially arose from the mismatch between the expectation and outcome (i.e., feedback). That is, the match or mismatch between distal intentions (i.e., to achieve a feedback of success) and an actual feedback (i.e., success or failure) likely influenced SoA even when the feedback was irrelevant to their task performance. Inoue et al. reported that SoA during continuous action was greater with better task performance even if the participant noticed that the better task performance was due to computer assistance [18]. However, the performance during the task and the final outcome were not dissociated in their study (i.e., better performance led to a better outcome). Thus, the present study could demonstrate that feedback of the final outcome retrospectively modulates SoA independently of performance during the continuous action task. This is likely related to the previous finding that SoA in simple action was retrospectively enhanced by positive outcome [23]. Therefore, the retrospective modulation of SoA is a more general effect and probably occurs for both simple discrete action and continuous action. Note that the present study might have a limitation as to the adopted question. Here, we simply asked participants how much they think that they controlled the white dot. This type of question might be interrupted differently among participants; some might report SoA based on whether the white dot was directed into the destination at the right point, and other might report SoA based on the only delay between key press and response of the dot. That said, as the present experimental paradigm requires participants to carry a white dot to a given target, SoA raised from directing the dot into the destination at the right point should strongly correlate with that from the response of the dot for each key press, and it is difficult to dissociate them. One reason for higher SoA with a shorter delay is that action-outcome interval is short. At the same time, another reason is that control of the dot to the destination direction becomes easier by the shorter delay. Therefore, we assumed that the participants reported the SoA based on both goal-directed inference and the responses of the dot, which highly correlated each other. However, as the delay was fixed in each trial, we assume that the SoA mostly arose from the goal-directed inference in the present experimental paradigm.

Effects of feedback on SoA have been investigated in a different paradigm [7,24]. In a study of Metcalfe and colleagues, participants played a computer game in which they tried to touch downward scrolling X’s by mouse control and avoided touching O’s [24]. When X was correctly touched, two types of explosion were prepared: 100% and 75%. In the 100% condition, explosion always occurred while in the 75% condition, explosion occurred with 75% of touches and nothing happened with 25% touches as if X was not touched. In Experiment 1, participants were asked SoA based on how many X’s they touched, not the number of explosions. Then, their results showed that the feedback (i.e., the number of explosions) significantly influenced SoA while the performance (i.e., touch rate) did not differ; the 100% explosion condition led to higher SoA. The main difference between Metcalfe et al. [24] and the present study was that participants in Metcalfe et al. could monitor the explosion feedback during their performances even if they were instructed to ignore the explosions (i.e., non-retrospective modulation) while participants in the present study did not receive any feedback such as success or failure until they carried the dot to the target. Taken together with the present results, SoA is generally influenced by feedback regardless of its timing of or attention to the feedback. In the present study, we did not instruct participants to ignore the feedback (success or failure). Hence, effects of instruction on the present experimental paradigm might need to be investigated in future studies.

Interestingly, the differences of SoA between the successful and failed trials were larger in the condition with more obstacles. Here, we would like to discuss a connection between the present results and self-serving bias [25], or the tendency for people to attribute causes of their positive outcomes more to themselves than external factors, and the opposite with negative outcome. In the present study, the enhanced SoA in the successful trials with more obstacles might occur because the participants attributed their success in this more difficult condition to their skills. On the other hand, the reduced SoA in the failed trials with more obstacles might be because the participants attributed their failures to the external causes (including the number of obstacles). Further investigations are needed to understand the relationship between SoA and self-serving bias. In addition, in the present study, we instructed to participants that an order key press might be sometimes ignored while the key press was always reacted because we tried to adopt the experimental procedure used in the previous study [8]. Therefore, the present instruction might lead participants to attribute unwanted consequences of their actions to external causes (e.g., ignorance of key press), which only occurred in failed trials. To investigate the effects of the possibly false attributions on SoA, another type of instruction in which the key press was always reacted needs to be adopted.

In the present study we were only able to measure SoA explicitly. Implicit measurements of SoA such as intentional binding [26] and sensory attenuation [11] have also been used. For example, intentional binding, in which subjective time perception is compressed between volitional action and an external effect, is not completely equivalent to measuring SoA by explicit questions (e.g., visual analog scale) but is instead regarded to reflect the implicit aspect of SoA [27]. Therefore, it would be theoretically interesting to examine implicit measures in continuous tasks as in the present study. However, there are several procedural difficulties. One was that, as continuous action tasks require a series of movements, if participants were asked to reproduce the delay duration after each trial such as in the intentional binding, their attention to the control of the dot might deviate and that to the timing likely increases [28]. For instance, Wen et al. adopted the dot control task as in previous studies [22]. In their experiment, 7 levels of delay (0–480ms) were prepared. After each trial, participants judged whether there was a delay between response and dot reaction. Wen et al. (2017) discussed that when participants answered only SoA, they put more attention to performance-based cues [22] while when they answered delay detection and SoA, they put more attention to sensory cues. Therefore, a paradigm including delay detection may have a possibility to measure implicit index of SoA, but the task demands modulate SoA in fact. Moreover, the number of key presses could not be controlled in the present paradigm, which also influences the reproduction of the delay duration (i.e., uneven experience). Finally, previous studies showed that the correlation between implicit and explicit indices of SoA was different when participants were asked to consider two indices simultaneously from when they were only asked to consider one in each trial [27], and in such a case the existence of correlation depended on the measurement method [5]. Hence, implicit measurement of SoA during continuous action is interesting to examine but an adequate method needs to be devised in further studies.

## Supporting information

S1 Table Individual information in each experimental condition. (XLSX)
